# Adrenalin Glycosuria

**Published:** 1914-05

**Authors:** 


**Adrenalin Glycosuria.** Anastazy Landau, Zeit. fuer Klin. Med., Vol .79, sections 3 and 4, verifies the belief that 150 grams of dextrose do not normally produce glycosuria, though the sugar is increased in the blood. The joint injection of adrenalin causes dextrosuria provided dextrose or a carbohydrae yielding it under digestion. The administration of levulose does not, however, cause levulosuria. Cocaine increases the action of adrenalin while opium diminishes it, by delaying the mobilization of glycogen and reducing the secretory activity of the kidney.

